# Glycosphingolipids of human embryonic stem cells

**DOI:** 10.1007/s10719-016-9706-y

**Published:** 2016-06-21

**Authors:** Michael E. Breimer, Karin Säljö, Angela Barone, Susann Teneberg

**Affiliations:** 1 Institute of Clinical Sciences, Department of Surgery, Sahlgrenska Academy at University of Gothenburg, Göteborg, Sweden; 2 Institute of Biomedicine, Department of Medical Biochemistry and Cell Biology, Sahlgrenska Academy at University of Gothenburg, P.O. Box 440, S-405 30 Göteborg, Sweden

**Keywords:** Human pluripotent stem cells, Human embryonic stem cells, Human induced pluripotent stem cells, Glycosphingolipid characterization, Mass spectrometry, Stem cell marker, Blood group antigens

## Abstract

The application of human stem cell technology offers theoretically a great potential to treat various human diseases. However, to achieve this goal a large number of scientific issues remain to be solved. Cell surface carbohydrate antigens are involved in a number of biomedical phenomena that are important in clinical applications of stem cells, such as cell differentiation and immune reactivity. Due to their cell surface localization, carbohydrate epitopes are ideally suited for characterization of human pluripotent stem cells. Amongst the most commonly used markers to identify human pluripotent stem cells are the globo-series glycosphingolipids SSEA-3 and SSEA-4. However, our knowledge regarding human pluripotent stem cell glycosphingolipid expression was until recently mainly based on immunological assays of intact cells due to the very limited amounts of cell material available. In recent years the knowledge regarding glycosphingolipids in human embryonic stem cells has been extended by biochemical studies, which is the focus of this review. In addition, the distribution of the human pluripotent stem cell glycosphingolipids in human tissues, and glycosphingolipid changes during human stem cell differentiation, are discussed.

## Background

The successful isolation of mouse embryonic stem cells [1, 2], and subsequently human embryonic stem cells (hESC) [3], from the inner cell mass of the blastocyst are milestones in regenerative medicine and life science. Takahashi’s and Yamanaka’s equally important contributions generating so-called induced pluripotent stem cells, initially from mouse [4], and thereafter from human [5] fibroblasts, by retrovirus-mediated transfection, expanded the field further. These findings theoretically enable new therapeutic alternatives and clinical applications based on an unlimited and renewable source of cells. Regardless of origin, both hESC and human induced pluripotent stem cells (hiPSC) possess the ability to be propagated indefinitely in culture and the capacity to generate cells of each of the three embryonic germ layers (endoderm, mesoderm and ectoderm), hence differentiate into any human cell type (reviewed in ref. [6]). Pluripotent stem cells (including both hESC and hiPSC) and their derivatives provide models for studying developmental biology and pathology, as well as for the development of novel drugs, toxicology screening, and in the future cell/organ transplantation therapies.

The increasing population of people worldwide suffering from chronic diseases and organ failure are great medical challenges. The successful derivation of insulin-producing β cells, cardiomyocytes, hepatocytes and neurons from pluripotent stem cells could eventually provide potential cures. However, there are many obstacles to overcome before stem cell therapy can be brought to the clinic. Pluripotent stem cells may not function normally *in vivo*, might retain tumorigenic potential, and the immune system of a recipient grafted with human stem cell derived cells/tissues will be challenged with non-self antigens which might lead to rejection, necessitating a life-long immunosuppressive therapy of the recipient. On the other hand, if hiPSC from the specific patient can be used, immune rejection may be eliminated.

Initially, human pluripotent stem cells were cultivated on mouse embryonic feeder cells, consequently potentially risking incorporation of animal components into the human cells, which further potentiates the immunogenicity of the grafts and aggravates the risk for rejection. In recent years technical development of media and culturing materials has made it possible to culture pluripotent cells and their derivatives under feeder- and xeno-free conditions [7]. This progress enables the production/development of Good-Manufacturing-Practice cell lines, and takes the stem cell research field closer to clinical practice.

## Embryonic stem cell markers

The rapid development in the stem cell field requires a parallel rapid progress in stem cell quality control, both of undifferentiated cells and cells after derivation and expansion. The expression of certain cell surface markers has been used for characterization since the first successful isolation of human embryonic stem cells [3]. Several of these stem cell markers are carbohydrates, as *e.g.* the tumor recognition antigens TRA-1-60 and TRA-1-81, and the stage-specific embryonic antigens SSEA-3 and SSEA-4 [8]. Recently, the blood group H type 1 epitope/SSEA-5 and the sialyl-lactotetra epitope were identified as novel carbohydrate markers of human pluripotent stem cells (hPSC) [9, 10]. The blood group H type 1 and the sialyl-lactotetra epitopes can be found on both glycoproteins and glycosphingolipids, whereas the globo-series determinants SSEA-3 and SSEA-4 have hitherto only been identified in glycosphingolipids.

However, although SSEA-3 and SSEA-4 are used as markers of undifferentiated hPSC, these glycosphingolipids are also present in some adult human tissues [11–13].

## Glycosphingolipids

In eukaryotic cells glycosphingolipids are predominantly found on the cell surface, with the lipophilic ceramide part located in the outer membrane leaflet and the carbohydrate part exposed to the surrounding environment [14]. The expression of glycosphingolipids varies both quantitatively and qualitatively between different species, individuals of the same species, organs and individual cells within an organ. The ceramide part consists of a fatty acid and a long-chain base, united by an amide linkage and a great number of molecular species results due to variations of the number of carbon atoms, double bonds, methyl branches and hydroxyl groups. The saccharide chain is attached, by a glycosidic linkage, to the primary hydroxyl group of the long-chain base. The size of the carbohydrate moiety normally ranges from 1 to 12 monosaccharide units, but glycosphingolipids with more than 30 saccharide residues (polyglycosylceramides) have been described. The oligosaccharide part exhibits a great complexity due to variation of the constituent monosaccharides, binding positions, glycosidic configuration, carbohydrate sequence and branching. When all the possible variations of the ceramide as well as the carbohydrate moiety are taken into account, an enormous potential structural complexity emerges [15]. More than 400 compounds are listed in a summary of identified glycosphingolipids [16].

Glycosphingolipids are divided into acid (negatively charged) and non-acid (neutral) components, where the acid glycosphingolipids are further divided into sulfate ester conjugated (sulfatides) and sialic acid containing structures (gangliosides). In addition, glycosphingolipids are classified on the basis of their carbohydrate core structures. In humans the lacto/type 1 (Galβ3GlcNAc), neolacto/type 2 (Galβ4GlcNAc), and globo/type 4 (Galα4Gal) core chains are the most common in non-acid glycosphingolipids, while gangliosides are mainly based on ganglio (Galβ3GalNAc) or neolacto core chains. The lacto and neolacto core chains are also present in glycoproteins, but the globo and ganglio core structures have hitherto only been identified in glycosphingolipids.

Several different isolation and analytical techniques are needed to achieve a complete structural characterization of glycosphingolipids from biological materials. Glycosphingolipids have to be isolated, and separated into non-acid components, gangliosides and sulfolipids, which thereafter need to be separated into individual molecular species [17]. Analytical techniques encompass mass spectrometry, NMR spectroscopy, chemical degradation and immunostaining [18]. To achieve this, substantial amounts of starting tissue material are required. When only small amounts of biological material are available, such as *in vitro* cultured cells and tissue biopsies, the isolation procedure has to be modified and analytical techniques restricted to immune assays and mass spectrometry. These simplified procedures usually eliminate certain glycosphingolipid species, and remaining non-glycosphingolipid contaminants hamper interpretation of the analytical data. Since cross-reactivity is a well-known phenomenon when using monoclonal antibodies directed against glycan epitopes [19], including the antibodies directed to SSEA-3 and Globo H [20], a cautious interpretation of the results obtained is necessary. Hence, the structural information gained is reduced, and there is an obvious risk of missing individual glycosphingolipids as well as confusing different structural components.

## Glycosphingolipid composition of hESC

In the first studies of hESC glycosphingolipids Liang *et al.* used flow cytometry, MALDI-MS and MS/MS to characterize glycosphingolipids from the upper phase obtained by Folch partition of crude lipid extracts [21, 22]. This allowed identification of non-acid glycosphingolipids of the globo series (globotetraosylceramide, globopentaosylceramide/SSEA-3 and the Globo H hexaosylceramide) and lacto series (type 1 core chain; lactotetraosylceramide and H type 1 pentaosylceramide). The gangliosides found were GM3, GM1, GD1a or GD1b, sialyl-globopentaosylceramide/SSEA-4 and di-sialyl-globopentaosylceramide. Glycosphingolipids identified and their structures are given in Table 1.

**Table 1:** Glycosphingolipids of human embryonic stem cells

No. trivial name	Structure	Methods^a^	References
*Simple compounds*			
1. Galactosylceramide^2^	Galβ1Cer	TLC, MS, NMR	[20]
2. Glucosylceramide	Glcβ1Cer	TLC, MS, NMR	[20]
3. Sulfatide	SO_3_-3Galβ1Cer	TLC, CBA, MS, IH, EM	[10]
4. LacCer	Galβ4Glcβ1Cer	TLC, MS, NMR	[20]
5. Sulf-LacCer	SO_3_-3Galβ4Glcβ1Cer	TLC, CBA, MS, IH, EM	[10]
6. Galabiaosylceramide	Galα4Galβ1Cer	TLC, MS, NMR	[20]
7. Lactotri	GlcNAcβ3Galβ4Glcβ1Cer	MS	[20, 23]
8. GM3	NeuAcα3Galβ4Glcβ1Cer	MS	[10, 21]
9. *GD3*	NeuAcα8NeuAcα3Galβ4Glcβ1Cer	MS	[10]
*Globoseries*			
10. Globotri	Galα4Galβ4Glcβ1Cer	MS, NMR	[20]
11. Globotetra	GalNAcβ3Galα4Galβ4Glcβ1Cer	MS, NMR	[20, 21, 23]
12. Globopenta/SSEA-3	Galβ3GalNAcβ3Galα4Galβ4Glcβ1Cer	CBA, MS, NMR, FC, IF	[20, 21, 23]
13. GloboH	Fucα2Galβ3GalNAcβ3Galα4Galβ4Glcβ1Cer	CBA, MS, NMR, FC, IF	[20, 21]
14. *Sialyl-globotetra* ^b^	NeuAcα3GalNAcβ3Galα4Galβ4Glcβ1Cer	MS	[10]
15. Sialyl-globopenta/SSEA-4	NeuAcα3Galβ3GalNAcβ3Galα4Galβ4Glcβ1Cer	CBA, MS, FC, IF	[10, 21, 23]
16. Disialyl-globopenta	NeuAcα3Galβ3(NeuAcα6)GalNAcβ3Galα4Galβ4Glcβ1Cer	MS	[10, 21]
17. *Sulf-globopenta*	SO_3_-3Galβ3GalNAcβ3Galα4Galβ4Glcβ1Cer	MS	[10]
*Lactoseries*			
18. Lactotetra	Galβ3GlcNAcβ3Galβ4Glcβ1Cer	MS, NMR, FC	[20–22]
19. H type 1 penta/SSEA-5	Fucα2Galβ3GlcNAcβ3Galβ4Glcβ1Cer	CBA, MS, NMR, FC	[20, 21, 23]
20. A type 1 hexa	GalNAcα3(Fucα2)Galβ3GlcNAcβ3Galβ4Glcβ1Cer	CBA, MS	[20]
21. Sialyl-lactotetra	NeuAcα3Galβ3GlcNAcβ3Galβ4Glcβ1Cer	CBA, MS, FC, IH, EM	[10]
*Neolactoseries*			
22. Neolactotetra	Galβ4GlcNAcβ3Galβ4Glcβ1Cer	CBA, MS	[20, 23]
23. *Neolactopenta*	HexNAc-Galβ4GlcNAcβ3Galβ4Glcβ1Cer	MS	[23]
24. *H type 2 penta*	Fucα2Galβ4GlcNAcβ3Galβ4Glcβ1Cer	MS	[20]
25. *Le* ^*x*^ *penta*	Galβ4(Fucα3)GlcNAcβ3Galβ4Glcβ1Cer	MS	[20]
26. Le^y^ hexa	Fucα2Galβ4(Fucα3)GlcNAcβ3Galβ4Glcβ1Cer	CBA, MS	[20, 23]
27. Le^x^ hepta	Galβ4(Fucα3)GlcNAcβ3Galβ4GlcNAcβ3Galβ4Glcβ1Cer	CBA, MS	[20]
28. Le^y^ octa	Fucα2Galβ4(Fucα3)GlcNAcβ3Galβ4GlcNAcβ3Galβ4Glcβ1Cer	CBA, MS	[20]
*Ganglioseries*			
30. NeuAc-GM1	Galβ3GalNAcβ4(NeuAcα3)Galβ4Glcβ1Cer	MS	[21, 22]
30. *NeuGc-GM1*	Galβ3GalNAcβ4(NeuGcα3)Galβ4Glcβ1Cer	MS	[23]
31. GD1a	NeuAcα3Galβ3GalNAcβ4(NeuAcα3)Galβ4Glcβ1Cer	MS	[10, 21]

^a^Methods used for characterization: TLC, thin-layer chromatography with chemical detection; MS, mass spectrometry; NMR, proton NMR; CBA, chromatogram binding assays; IH, immunohistochemistry; EM, immune electron microscopy, FC, flow cytometry, IF, immunofluorescence

^b^Glycosphingolipids in italics have been characterized in only one study and by only one method

Glycosphingolipids were also characterized by MALDI-TOF/TOF MS of oligosaccharides released from glycosphingolipids by enzyme treatment in a highly ambitious study of the total cellular glycome (glycosaminoglycans, *N*- and *O*-linked saccharides, and glycosphingolipids) of human pluripotent stem cell lines (hESC and hiPSC) [23]. However, in this study the cells were cultured on mouse feeder cells. Culture of hESC in the presence of animal-derived compounds may lead to uptake of compounds from the culture medium, as *e.g.* the nonhuman sialic acid NeuGc [24], and feeder cells may also have contaminated the human stem cell preparations. Thus, the interpretation of data was challenging in this case. After eliminating glycan structures that were probably of mouse origin, it was concluded that compared to human somatic cells, the human stem cells had a higher expression of the glycosphingolipids globopentaosylceramide/SSEA-3, sialyl-globopentaosylceramide/SSEA-4, fucosyl-lactotetraosylceramide/SSEA-5, difucosyl-neolactotetraosylceramide, neolactopentaosylceramide, neolactotetraosylceramide, globotetraosylceramide, the NeuGc-GM1 ganglioside, and gangliotriaosylceramide. Thus, the list of characterized stem cell glycosphingolipids still contains some compounds that are likely to be contaminants from the feeder cells or culture medium, as *e.g.* the non-human ganglioside NeuGc-GM1.

In order to allow characterization of minor glycosphingolipids of hESC and identify novel stem cell markers, glycosphingolipids from two hESC lines (SA121, SA181) were isolated using a large number of cells (1x10^9^ cells per cell line) as starting material [10, 20]. These cells were grown without animal derived material in the culture medium [7]. After chloroform/methanol extraction and mild alkaline methanolysis, followed by repeated column chromatographies of both native compounds and acetylated derivatives, approximately 2.5 mg of total acid glycosphingolipids and 3 mg of total non-acid glycosphingolipids were obtained from each cell line. After initial structural characterization, the total acid and total non-acid fractions were separated on silicic acid columns into partly purified sub-fractions, allowing a further characterization of minor glycosphingolipid compounds. Structural determination was carried out with complementary techniques, including thin-layer chromatogram binding assays, LC-ESI/MS of native glycosphingolipids and glycosphingolipid-derived oligosaccharides, and proton NMR spectroscopy. In addition, the cellular distribution of selected carbohydrate structures was studied by flow cytometry, immunohistochemistry and immune electron microscopy. By combining the data obtained by these analytical methods, a complex expression of variant glycosphingolipids was found, and several hESC glycosphingolipids, not previously described, were identified (Table 1) [10, 20]. Thus, in addition to the globo series and type 1 core chain glycosphingolipids previously characterized, a number of non-acid type 2 core chain glycosphingolipids (neolactotetraosylceramide, the H type 2 pentaosylceramide, the Le^x^ pentaosylceramide, and the Le^y^ hexaosylceramide) were identified, and also the blood group A type 1 hexaosylceramide. Novel acid hESC glycosphingolipids identified were sulfatide, sulf-lactosylceramide, sulf-globopentaosylceramide, sialyl-globotetraosylceramide and sialyl-lactotetraosylceramide.

Furthermore, a number of short chain glycosphingolipids were identified as glucosylceramide, galactosylceramide, lactosylceramide, galabiaosylceramide, lactotriaosylceramide and globotriaosylceramide. These compounds, along with part of the more polar ones (4–6 carbohydrate residues), are retained in the lower phase after Folch partitioning. Therefore, in studies using organic solvent/water partition as part of the isolation procedure [21, 22], these glycosphingolipids are not included. For the negatively charged gangliosides, the major part of the short chain compounds (≥3 carbohydrate residues) is found in the upper phase.

## Acid glycosphingolipids of hESC

### Sulfated glycosphingolipids

Analyses of the acid glycosphingolipids of hESC by antibody binding and LC-ESI/MS identified sulfatide and sulfated lactosylceramide [10]. Sulfatide, and to some extent sulf-lactosylceramide, are present in several normal human tissues, as *e.g.* brain, kidney, gastro-intestinal tract including pancreas, and trachea (reviewed in ref. [25]).

Sulfated glycosphingolipids were not found in the studies of hESC glycosphingolipids by Liang *et al.* [21, 22]. This is mostly likely due to the use of permethylated glycosphingolipid derivatives in the mass spectrometric analysis, since sulfate groups are lost upon permethylation. In a similar manner, sulfatide was not reported in the study by Fujitani *et al.*, since MALDI-TOF mass spectrometry in the positive ion mode was used for characterization of the glycosphingolipid-derived oligosaccharides [23].

No sulfatide/sulf-lactosylceramide was detected on the cell surface of hESC using the anti-SO_3_-3Galβ antibody in flow cytometry, and immunohistochemistry showed only a faint cytoplasmic staining. Furthermore, immune electron microscopy with the anti-sulfatide antibody O4 gave no staining of the hESC cell surface, whereas a distinct staining of mitochondria and Golgi, was obtained. Sulfatide is a marker of immature oligodendrocytes [26], and the binding of this sulfatide recognizing antibody O4 is used to monitor hESC and hiPSC differentiation into oligodendrocytes, where it is detected after four weeks of differentiation [27]. Interestingly, our findings demonstrate that sulfatide is produced already in undifferentiated hESC, but is retained within intracellular compartments. Thus, upon oligodendrocyte differentiation there is a change in the subcellular distribution of sulfatide, which should be further investigated.

### Sialyl-lactotetraosylceramide

**Fig. 1:**
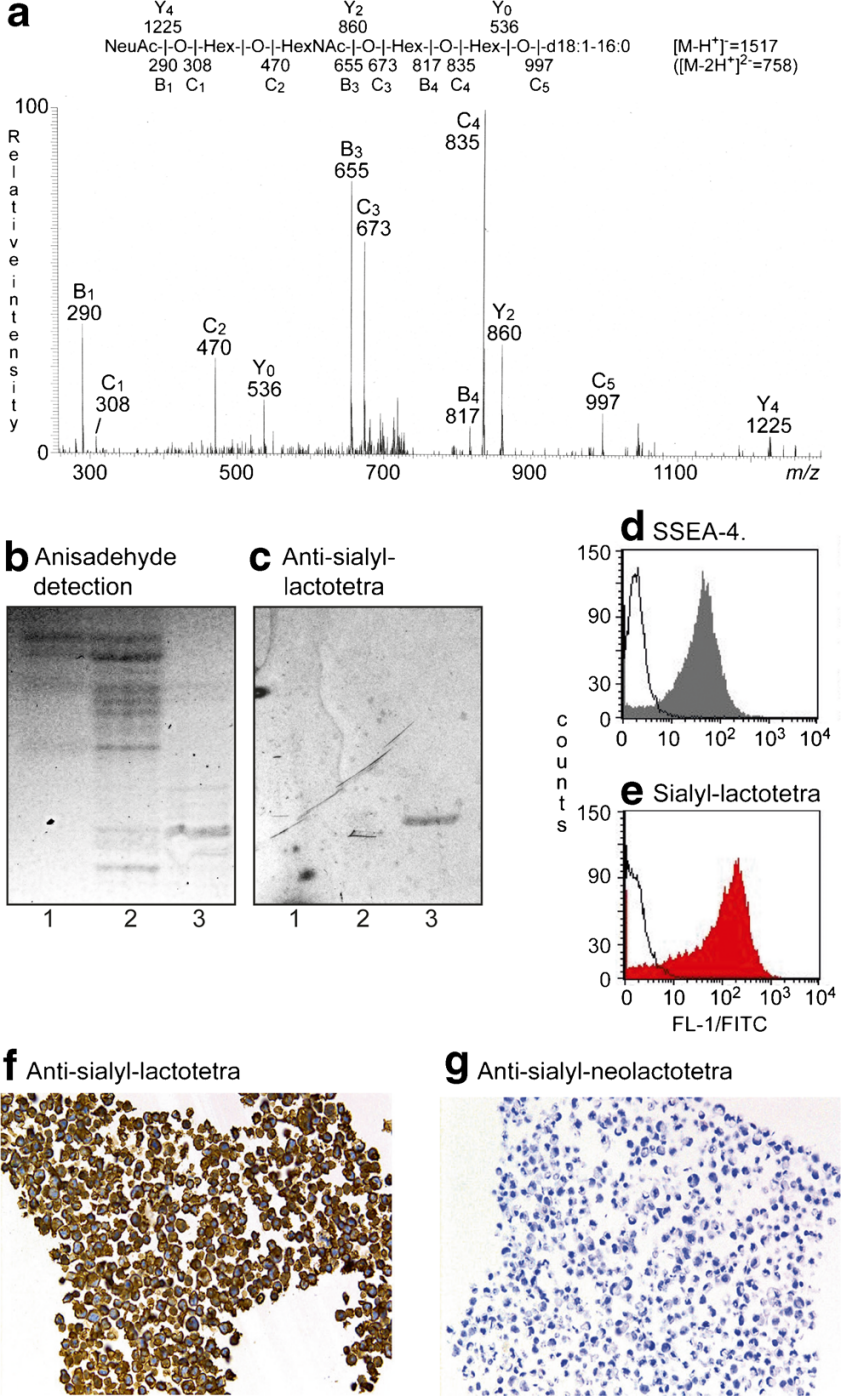
Studies of the sialyl-lactotetra glycosphingolipid in hESC. (**a**) MS^2^ spectrum of the [M-2H^+^]^2−^ ion at *m/z* 758 from LC-ESI/MS of the hESC ganglioside fraction 181C. The [M-2H^+^]^2−^ ion at *m/z* 758 corresponds to a [M-H^+^]^−^ ion at *m/z* 1517, and indicates a ganglioside with one NeuAc, one HexNAc, three Hex, and d18:1–16:0 ceramide. MS^2^ of the ion at *m/z* 758 gave spectrum with B- and C-type fragment ions (B_1_ at *m/z* 290, C_1_ at *m/z* 308, C_2_ at *m/z* 470, B_3_ at *m/z* 655, C_3_ at *m/z* 673, B_4_ at *m/z* 817, C_4_ at *m/z* 835 and C_5_ at *m/z* 997), which together with the Y_0_ ion at *m/z* 536, demonstrated a glycosphingolipid with NeuAc-Hex-HexNAc-Hex-Hex carbohydrate sequence and d18:1–16:0 ceramide. Cross-ring ^0,2^A-type fragments are diagnostic for carbohydrates substituted at C-4 [28]. The absence of a ^0,2^A_3_ fragment ion at *m/z* 572 indicated a 3-substituted HexNAc. (**b**) Thin-layer chromatogram stained by the chemical reagent anisaldehyde, and (**c**) immunostained using the anti-sialyl-lactotetra antibody. Lane 1, hESC SA181 acid glycosphingolipid fraction 181A, Lane 2, hESC SA181 acid glycosphingolipid fraction 181B; Lane 3, hESC SA181 acid glycosphingolipid fraction 181C. (**d**) Flow cytometry diagram of hESC incubated with antibodies against SSEA-4 and (**e**) sialyl-lactotetra. (**f**) Immunohistochemistry of hESC using anti-sialyl-lactotetra and (**g**) anti-sialyl-neolactotetra antibodies

Sialyl-lactotetraosylceramide was characterized as a hESC glycosphingolipid [10] by mass spectrometry (Fig. 1a), and binding of anti-sialyl-lactotetra antibodies on thin-layer chromatograms (Fig. 1c). No binding of anti-sialyl-neolactotetra antibodies to the acid glycosphingolipids of hESC was obtained.

A high cell surface expression of sialyl-lactotetra on both hESC and hiPSC was demonstrated by flow cytometry (Fig. 1e), immunohistochemistry (Fig. 1f), and immune electron microscopy. Indeed, all seven hESC lines and three hiPSC lines analyzed showed distinct cell surface anti-sialyl-lactotetra staining by immunohistochemistry, whereas no staining with anti-sialyl-neolactotetra was observed (Fig. 1g). However, there was no staining of hESC-derived hepatocyte-like or cardiomyocyte-like cells. Upon differentiation into hepatocyte-like cells the sialyl-lactotetra epitope was rapidly down-regulated and not detectable after 14 days. The loss of sialyl-lactotetra upon differentiation occurred in parallel with the reduced expression of the established pluripotency markers SSEA-4 and TRA-1-60. Expression of the sialyl-lactotetra epitope is also decreased in neuronal stem cells differentiating from hiPSC (K. Säljö *et al.*, in manuscript). Taken together these findings identify sialyl-lactotetra as a promising new marker of undifferentiated human pluripotent stem cells.

Sialyl-lactotetraosylceramide has not been identified in adult human tissues, but is present in the brains of children under the age of two [29], as well as in human meconium [30], the first stool of the newborn consisting mainly of extruded mucosal cells from the developing fetal gastrointestinal tract, indicating that sialyl-lactotetraosylceramide is produced during the development of the gastrointestinal tract.

### Sulf-globopentaosylceramide and sialyl-globotetraosylceramide

Sulf-globopentaosylceramide and sialyl-globotetraosylceramide are also potential novel markers of human pluripotent stem cells. These hESC glycosphingolipids were characterized by mass spectrometry [10], and should be confirmed by other analytical methods. Sulf-globopentaosylceramide has hitherto only been identified in adult human kidneys [31]. Sialyl-globotetraosylceramide has not been reported in normal human tissues, but has been characterized in human teratocarcinoma cells [32], and in muscles affected by amyothropic lateral sclerosis [33]. Terminal NeuAcα3GalNAc is a rare sequence, but is found in the sialyl-*x*_2_ glycosphingolipid (NeuAcα3GalNAcβ3Galβ4GlcNAcβ3Galβ4Glcβ1Cer; [34]), and α2,3-sialylation of the terminal GalNAc of *x*_2_ and globotetraosylceramide by the α2,3-sialyltransferase ST3Gal II has been demonstrated [35].

Development of specific reagents as *e.g.* monoclonal antibodies specific for sulf-globopentaosylceramide and sialyl-globotetraosylceramide are urgently needed for studies of the expression of these compounds during hPSC differentiation.

## Non-acid glycosphingolipids of hESC

### Blood group AB(O)H antigens

The ABO histo-blood group system is the strongest histocompatibility antigen system in human allotransplantation. Even if successful ABO incompatible live donor renal allotransplantation protocols have been introduced during the last decade [36], this immunological barrier is still of significant importance. Clinical applications grafting hESC, and/or tissues derived from these cells, will challenge the immune system of the recipient with non-self antigens. Therefore, ABO typing of hESC is important, and cell lines of blood group type O should preferentially be used. A few years ago, we showed for the first time that hESC lines expressed blood group A/B antigens in concordance with the cells ABO genotypes [37]. There was also a slightly different expression of A and B antigens, with B antigen mainly on the cell surface, while A antigens were found both on the cell surface and in the cytoplasm. These cell lines were cultured on mouse feeder fibroblasts and cell amounts available were too small to allow any chemical characterization.

A great structural diversity of blood group AB(O)H glycosphingolipids are found in adult tissues/cells both regarding number of carbohydrate residues and type of core chains [38, 39]. Therefore a very interesting finding was that the two hESC lines, both of blood group A_1_ genotype, expressed the A antigen determinant only as a type 1 core chain hexaglycosylceramide [20]. This is in contrast to the type 2 core chain based Le^x^ and Le^y^ determinants, which were found on glycosphingolipids with different chain lengths [20].

Blood group H type 1 pentaosylceramide was the major blood group compound in the two hESC lines in our study [20]. The H type 1 chain determinant has been identified as a hPSC marker denoted SSEA-5 [9], and recently a monoclonal antibody specific for the H type 1 pentaosylceramide was generated by immunization with hiPSC, and shown to exhibit potent dose-dependent cytotoxicity to the hiPSC [40]. The H type 1 pentaosylceramide was earlier identified in human meconium [41], and also in meconium from a 17 weeks old fetus [42]. However, blood group H type 1 chain glycosphingolipids are not specific for fetal tissues, since these compounds are present in large amounts in adult human tissues, predominantly in endodermal derived glandular epithelial tissues like pancreas [43] and intestinal epithelium [38, 44]. In contrast, mesodermal-derived organs lack, or have only small amounts of type 1 chain blood group antigens [38].

### Lewis and related antigens

The type 1 chain based Le^a^ and Le^b^ antigens are present in large amounts in human tissues, similar to the blood group ABH antigens described above [38]. These Lewis antigens were identified in human fetuses from 37 mm of length [45], and are present in large amounts in meconium [41]. Still Le^a^ or Le^b^ glycosphingolipids have not been identified in any of the hESC lines characterized [20–23], and the Le^a^ or Le^b^ determinants are not detected by flow cytometry of several other hESC and hiPSC lines (K. Säljö *et al.*, in manuscript). This is intriguing since only 20 % of Caucasians are Lewis negative.

The corresponding type 2 core chain glycosphingolipids Le^x^ and Le^y^ were identified in the investigated hESC lines [20]. Immunohistochemistry studies show that in human embryos the expression of Le^x^ and multimeric Le^x^ peaks at specific developmental stage, mostly at 40–80 days, followed by a decline upon further development [46]. Both compounds are mainly found in gastrointestinal and urogenital epithelia, but the tissue distribution of Le^x^ is wider than for multimeric Le^x^. Miyake *et al.* demonstrated a variation in expression of the Le^x^, Le^y^ and sialyl-Le^a^ antigens during lung development of human embryos [47]. Furthermore, the Le^x^ and Le^y^ determinants have been found in adult human organs by immunohistochemistry (reviewed in ref. [48]), and chemically identified in glycoproteins [49] and glycosphingolipids [38, 50].

### Are SSEA-1 and Le^x^ identical antigen structures?

The presence of Le^x^ sequences in undifferentiated hESC is a controversial and confusing issue. The confusion is, at least partly, caused by the marketing of the SSEA-1 defining antibody MC480 [51] as an antibody specific for the Le^x^ determinant as well as a differentiation marker. However, the antibody MC480 reactivity is restricted to the Le^x^ epitope presented on long type 2 core chains, as *e.g.* among the slow-migrating non-acid glycosphingolipids of human erythrocytes [50, 52].

No binding of the MC480 antibody to hESC is found by immunohistochemistry or flow cytometry, indicating that SSEA-1 is not expressed in undifferentiated hESC. However, SSEA-1 is expressed upon initiation of cell differentiation [3, 53]. The fact that the MC480/SSEA-1 antibody does not bind to undifferentiated hESC has led to the interpretation that there are no Le^x^ glycoconjugates in these cells.

**Fig. 2:**
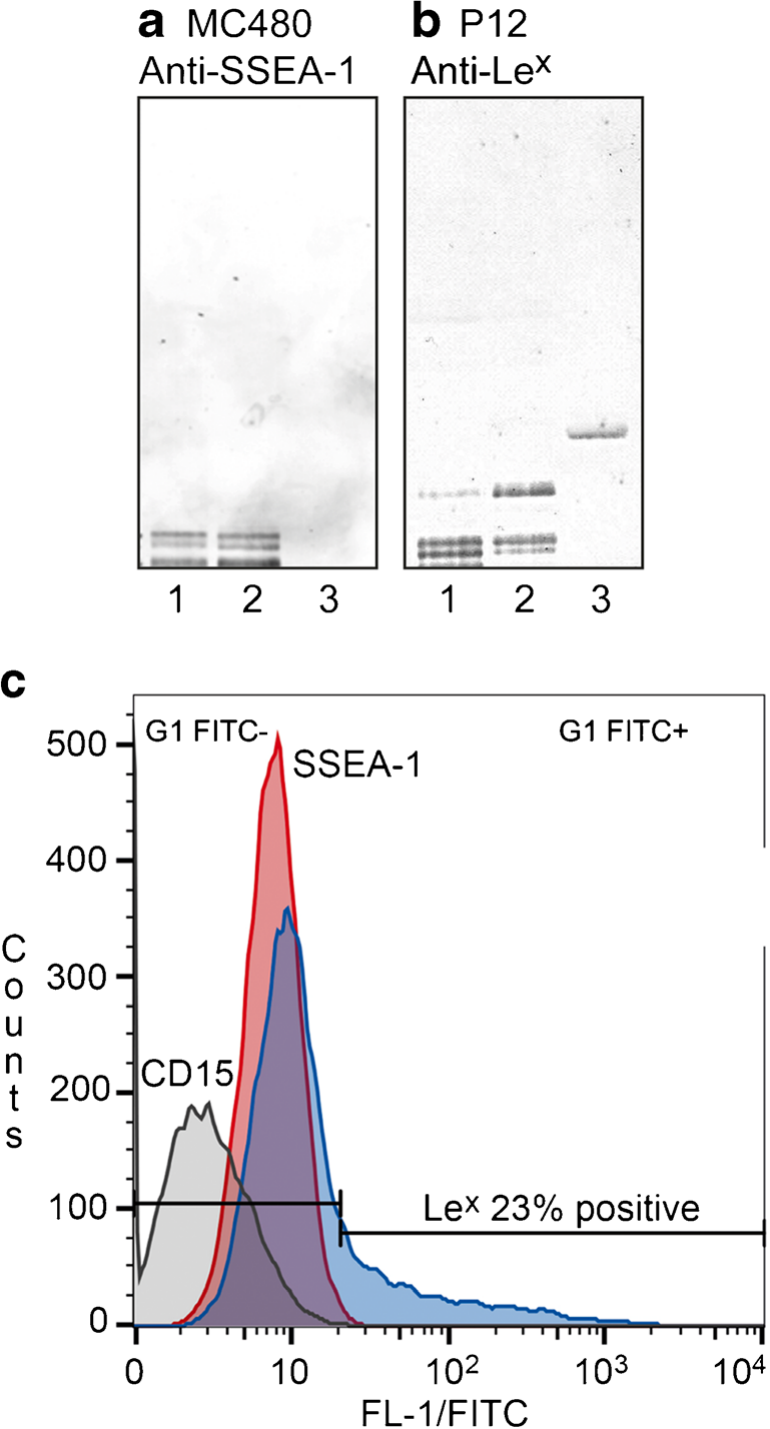
Binding of anti-SSEA-1 monoclonal antibody MC480 (**a**) and anti-Le^x^ monoclonal antibody P12 (**b**) to non-acid glycosphingolipids of undifferentiated hESC. Lane 1, hESC SA181 non-acid glycosphingolipid fraction IV; Lane 2, reference total non-acid glycosphingolipids of human blood group AB erythrocytes; lane 3, reference Le^x^ pentaosylceramide. (**c**) Flow cytometry diagram of hiPSC incubated with antibodies against CD15 (C3D-1; *grey*), SSEA-1 (MC480; *red*), and Le^x^ (P12; *blue*)

However, in the non-acid glycosphingolipid fractions of undifferentiated hESC the MC480/SSEA-1 antibodies react with several slow-migrating glycosphingolipids present in very small amounts (Fig. 2a, lane 1). The anti-Le^x^ antibody P12, has the capacity to bind to the Le^x^ pentaosylceramide (Fig. 2b, lane 3), and also binds in the heptaosylceramide region and to minor slow-migrating glycosphingolipids of the undifferentiated hESC (Fig. 2b, lane 1). Furthermore, when undifferentiated hESC were analyzed by flow cytometry, the P12 antibody gave distinct cell reactivity, while the MC480 antibody was negative (Fig. 2c). Taken together the mass spectrometry and antibody binding data thus demonstrate that glycosphingolipids with terminal Le^x^ sequences are present in undifferentiated hESC. The Le^x^ determinant has also been characterized in hESC *N*-glycans [54]. Short chain Le^x^ epitopes recognized by the P12 antibody are readily detectable on the cell surface, whereas Le^x^ epitopes on long type 2 core chains recognized by the MC480/SSEA-1 antibody are not. This might be due to the very low amounts of these MC480 binding compounds, or that these glycosphingolipids reside in intracellular compartments and have not yet reached the cell surface. Up-regulation of SSEA-1 expression upon differentiation should thus correspond to an increased level of cell surface glycoconjugates with terminal Le^x^ determinants on long poly-*N*-acetyllactosamine chains.

**Fig. 3:**
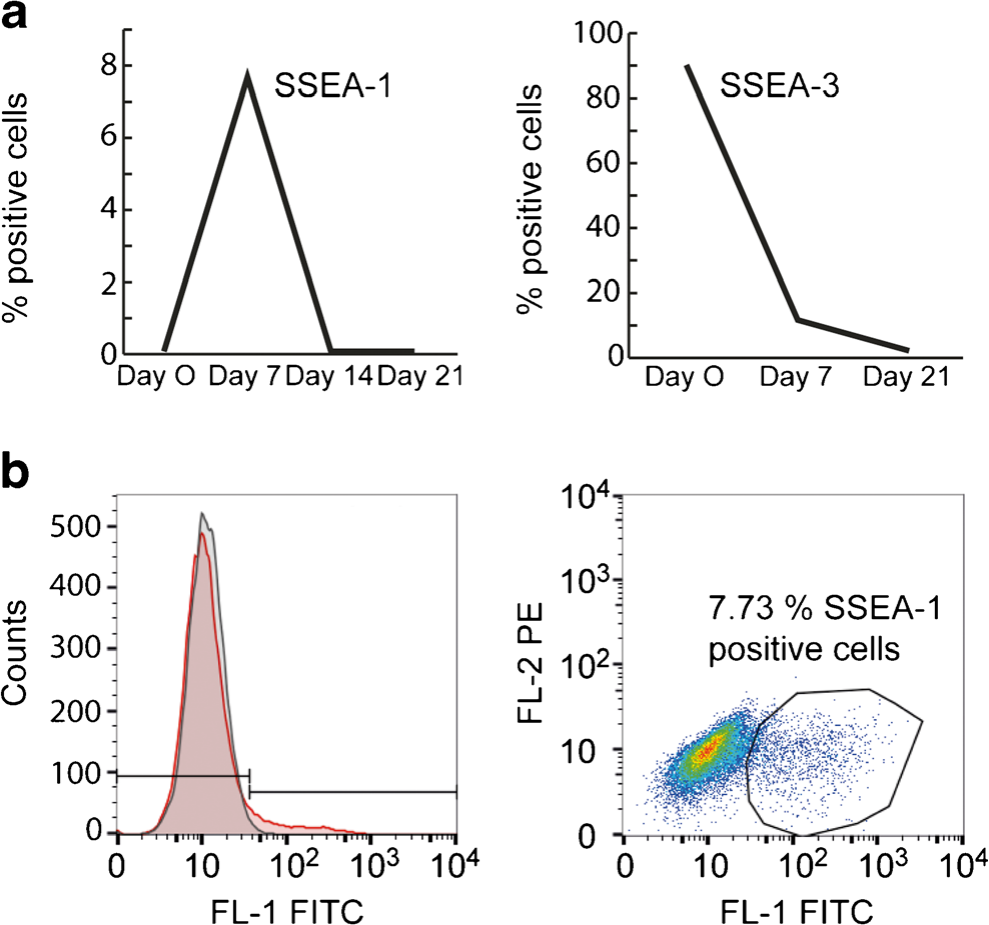
(**a**) Flow cytometry analysis of cell surface expression of SSEA-1 (clone MC480) and SSEA-3 (clone MC631) on hESC line SA121 during differentiation from hESC into hepatocyte-like cells (day 0, 7, 14 and 21). (**b**) Flow cytometry diagram of hESC line SA121 incubated with antibodies against SSEA-1 (MC480; *red*) on day 7 after induction of differentiation into hepatocyte like cells. Approximately 8 % of the cells transiently express SSEA-1 on day 7

Since the beginning of stem cell research, the expression of SSEA-1 has been considered to be a marker of differentiation in human stem cells [3, 53]. However, in the cell lines analyzed by us an increased expression of SSEA-1 is only observed during a short period after induction of differentiation, as illustrated by the quite modest and transient increase of SSEA-1 (clone MC480) expression during differentiation from hESC into hepatocyte-like cells. Only a distinct subpopulation (approximately 8 %) of the cells express SSEA-1 on their cell surface on day 7 after induction of differentiation (Fig. 3). A similar transient increase of SSEA-1 was observed during neuronal differentiation of hESC [55]. SSEA-1 was expressed during the neural stem cell stage (days 18–23 *in vitro*), while more differentiated neural precursor cells (days 30–37 *in vitro*) were SSEA-1 negative. Therefore, in humans SSEA-1 seems to be a differentiation marker for an early transient period, and not a general differentiation marker.

## Ceramide composition of hESC glycosphingolipids

Sphingosine (d18:1) with non-hydroxy 16:0 fatty acid is the main ceramide species of hESC glycosphingolipids, as determined by mass spectrometry [10, 20, 21]. In addition there are minor species with sphingosine and non-hydroxy 18:0–24:0/24:1 fatty acids, and among the sulfatides sphingosine with hydroxy 16:0 and 18:0 fatty acids are also present. Thus, the ceramide composition of hESC glycosphingolipids is relatively simple in contrast to the structural diversity of ceramides found in adult human tissues [14]. In *e.g.* adult human brain gangliosides the major ceramides are d18:1–18:0 and d20:1–18:0, whereas the epithelial cells of the human small intestine have predominantly phytosphingosine (t18:0) with hydroxy 16:0–24:0 fatty acids.

## Changes in hESC carbohydrate antigen expression upon differentiation

The changes in hESC surface antigens upon differentiation induced by retinoic acid, hexamethylene bisacetamide and dimethylsulfoxide was studied by flow cytometry by Draper *et al.* [56], demonstrating a rapid decrease in the expression of SSEA-3 and SSEA-4 following all types of induction. There was also an increased expression of several gangliosides, as *e.g.* GT3 and 9-O-acetyl-GD3. A transient increase in the expression of SSEA-1 was obtained by retinoic acid induction, but not with the other agents.

Similar patterns, *i.e.* decreased expression of globo-series (SSEA-3 and Globo H) and lacto-series (lactotetraosylceramide and H type 1 pentaosylceramide) glycosphingolipids, and increased expression of the gangliosides GM3, GM1 and GD3, were observed upon differentiation of hESC toward neural progenitor cells [22]. In contrast, a high expression of the globo-series (mainly globotetraosylceramide) and lacto-series glycosphingolipids remained after differentiation into endodermal cells, and in this case there was no increase in ganglioside expression. The expression of SSEA-5 and sialyl-lactotetra is also rapidly reduced in differentiating cells [9, 10].

Studies on changes in cell surface glycan distribution in the early development of human embryos are still very limited. In the early 1960’s, Szulman described ABH and Lewis antigen expression in a large number of fetuses from 5 to 14 weeks gestation age using immunohistochemistry [45, 53]. An interesting variation, depending on the fetus age, was demonstrated both regarding the cell wall located and excreted ABH antigens. It was also shown that certain tissues/cells of the fetuses, as *e.g.* cardiomyocytes and hepatocytes of 18–24 mm human embryos, did not express ABH antigens early in the development [57]. This is in accordance with our finding that a blood group B hESC line lost the B antigen expression upon differentiation into cardiomyocyte like cells [37]. In the adult human heart and liver, both cardiomyocytes and hepatocytes completely lack ABH antigens, while vascular endothelium and bile ducts do express ABH antigens. In a more recent study, Henderson *et al.* showed by immunohistochemistry on human embryos that SSEA-3 and SSEA-4 are found in the inner cell mass of expanded or hatching blastocysts, whereas SSEA-1 is present in trophectoderm [53].

## Final remarks

In recent years it has been possible to characterize the structures of human embryonic stem cell glycosphingolipids by biochemical methods. The first studies of hESC glycosphingolipids from crude lipid extracts [21, 22] allowed identification of the major compounds of these cells as based on globo core chains (globotetraosylceramide, globopentaosylceramide/SSEA-3, Globo H hexaosylceramide, sialyl-globopentaosylceramide/SSEA-4 and di-sialyl-globopentaosylceramide), and type 1 core chains (lactotetraosylceramide and fucosyl-lactotetraosylceramide/H type 1 pentaosylceramide). In addition the gangliosides GM3, GM1, GD1a or GD1b were found. A similar profile was described in a recent study of hiPSC glycosphingolipids [58]. Starting from considerably more hESC material, glycosphingolipid fractions could be isolated [10, 20] and, in addition to the major compounds, it was also possible to identify minor glycosphingolipids, as *e.g.* type 2 core chain glycosphingolipids and even some very rare minor acid glycosphingolipids (sialyl-globotetraosylceramide and sulf-globopentaosylceramide).

A remaining question is whether the carbohydrate expression of undifferentiated cells changes as a result of differentiation, or if glycans influence or play a more direct role in cellular destiny. The role of SSEA-3 and SSEA-4 glycosphingolipids in maintaining hESC pluripotency has been investigated by blocking glycosphingolipid biosynthesis in hESC using the inhibitors D-PDMP or ISP-1 (myriocin) [59]. hESC depleted of glycosphingolipids still differentiated to ectodermal, endodermal, and mesodermal lineages *in vivo*, and the gene expression profile of the cells was not altered, demonstrating that glycosphingolipids do not have a critical function in the maintenance of the undifferentiated state of hESC. However, when embryoid bodies were generated from hESC in the presence or absence of inhibitors, the gene expression profile of the embryoid bodies from hESC depleted of glycosphingolipids was distinctly different from the profile of control embryoid bodies, suggesting a role for glycosphingolipids during cellular differentiation. Interestingly, targeted disruption of the *Ugcg* gene encoding glucosylceramide synthase in mice gave embryos that underwent implantation and at first appeared to develop normally [60]. Gastrulation was initiated in the mutant embryos with differentiation into embryonic germlayers. However, at this stage an apoptotic process resulted in embryonic death. Furthermore, the capacity of embryonic stem cells with disrupted *Ugcg* gene to differentiate *in vivo* was severely impaired. Taken together these studies demonstrate a role for glycosphingolipids during embryonic development and differentiation of tissues, possibly by influencing critical cell-cell and cell-matrix interactions.

An important aspect in hPSC-derived cell replacement therapy is the risk of tumorigenesis from residual undifferentiated cells in the differentiated cell cultures [61]. Thus, sensitive assays to detect residual hPSC contamination in the differentiated cell population and methods to eliminate contaminating undifferentiated hPSC, are needed. Cell surface carbohydrate markers of hPSC can be important tools for the selective removal of undifferentiated hPSC [62]. The range of such carbohydrate markers has now been extended by the creation of the monoclonal antibody against the H type 1 glycosphingolipid with potent dose-dependent cytotoxicity to hiPSC [40], which may be used for elimination of undifferentiated cells. The recently identified sialyl-lactotetra marker of undifferentiated hPSC [10] may also be used for negative selection of undifferentiated stem cells in therapeutic cell products.

### Abbreviations

The glycosphingolipid nomenclature follows the recommendations by the IUPAC-IUB Commission on Biochemical Nomenclature (CBN for Lipids: *Eur. J. Biochem*. (1998) 257, 293). It is assumed that Gal, Glc, GlcNAc, GalNAc, NeuAc and NeuGc are of the D-configuration, Fuc of the L-configuration, and all sugars are present in the pyranose form.

In the shorthand nomenclature for fatty acids and bases, the number before the colon refers to the carbon chain length and the number after the colon gives the total number of double bonds in the molecule. Fatty acids with a 2-hydroxy group are denoted by the prefix h before the abbreviation *e.g.* h16:0. For long chain bases, d denotes dihydroxy and t trihydroxy. Thus d18:1 designates sphingosine (1,3-dihydroxy-2-aminooctadecene) and t18:0 phytosphingosine (1,3,4-trihydroxy-2-aminooctadecane).

EB, Embryoid bodies; hESC, Human embryonic stem cells; hiPSC, Human induced pluripotent stem cells; hPSC, Human pluripotent stem cells; LC-ESI/MS, Liquid chromatography-electrospray ionization mass spectrometry; SSEA, Stage-specific embryonic antigen.
